# Crystal structure of tetra­hydro­seselin, an angular pyran­ocoumarin

**DOI:** 10.1107/S205698901700932X

**Published:** 2017-07-04

**Authors:** A. K. Bauri, S. Foro, A. F. M. M. Rahman

**Affiliations:** a Bio-Organic Division, Bhabha Atomic Research Centre, Trombay, Mumbai 400 085, India; b Institute of Materials Science, Darmstadt University of Technology, Alarich-Weiss-Strasse 2, D-64287 Darmstadt, Germany; c Department of Applied Chemistry & Chemical Engineering, University of Dhaka, Dhaka 1000, Bangladesh

**Keywords:** crystal structure, naturally occurring seselin, *T. stictocarpum*, tetra­hydro­seselin, Pd/C hydrogenation in MeOH

## Abstract

In the title compound, tetra­hydro­seselin, C_14_H_16_O_3_, a pyran­ocoumarin [systematic name: 8,8-dimethyl-3,4,9,10-tetra­hydro-2*H*,8*H*-pyrano[2,3-*f*]chromen-2-one] obtained from the hydrogenation of seselin in the presence of Pd/C in MeOH at room temperature, the dihedral angle between the central benzene ring and the best planes of the outer fused ring systems are 6.20 (7) and 10.02 (8)°. In the crystal, mol­ecules show only very weak inter­molecular C—H⋯O inter­actions.

## Abstract 2

The title compound, tetra­hydro­seselin (THS), a hydrogenated product of the angular pyran­ocoumarin seselin, possesses photo-biological activity against different kinds of inflammatory skin diseases such as atopic dermatitis and pigment disorders like vitiligo and psoriasis.

## Chemical context   

The title mol­ecule, tetra­hydro­seselin, a hydrogenated product of an angular pyran­ocoumarin, seselin, consists of three different kinds of fused rings: a central benzene ring, an outer pyrone ring and a pyrane ring with dimethyl substituents attached at C3. These pyran­ocoumarins have absorption bands in the near UV region resulting from the presence of conjugated double bonds in the enone system and exhibit photo-mutagenic and photo-carcinogenic properties (Appendino *et al.*, 2004 ▸), which bind with the purine base of DNA in living cells to yield photo-adducts (Conforti *et al.*, 2009 ▸). Based on this property, the mol­ecules are used to treat numerous inflammatory skin diseases such as atopic dermatitis and pigment disorders like vitiligo and psoriasis, through exposure to UV radiation in photo dynamic therapy (PDT). Because of their strong ability to absorb UV radiation, these classes of mol­ecules are utilized as photo-protective agents to prevent the absorption of harmful UV radiation by the skin, in the form of a variety of sun-screening lotions widely used in dermatological applications in the cosmetic and pharmaceutical industries (Chen *et al.*, 2007 ▸, 2009 ▸). Also, *in vitro* anti-proliferative activity and *in vivo* photo-toxicity against numerous cancer cell lines, *e.g.* HL60 and A431, has been observed (Conconi *et al.*, 1998 ▸). In addition, this class of coumarins have been successfully used in the treatment of inhibited proliferation in the human hepatocellular carcinoma cell line (March *et al.*, 1993 ▸). Experimental results have shown that its photo-toxicity is extended *via* a Diels–Alder reaction to bind the double bond of a purine base of DNA in the living cell with the double bonds of coumarin to yield mono [(2 + 2) cyclo­addition] and diadducts [(4 + 2) cyclo­addition] (Conforti *et al.*, 2009 ▸). As a part of our studies in this area, we are looking at the role of double bonds in the photo-biological activity of the aforesaid mol­ecule. The crystal structure of the title compound tetra­hydro­seselin, C_14_H_16_O_3_, is reported herein.
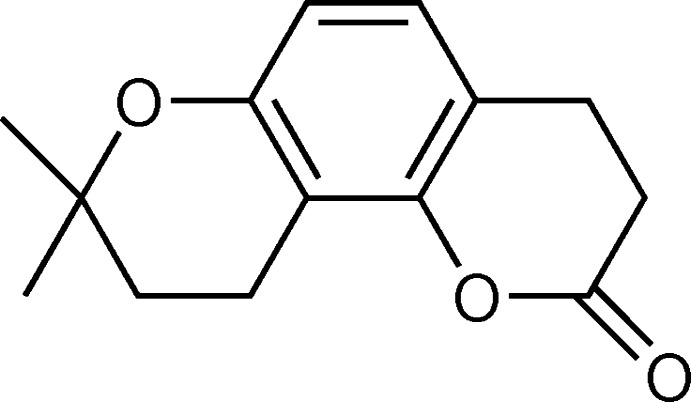


## Structural commentary   

**Figure 1:**
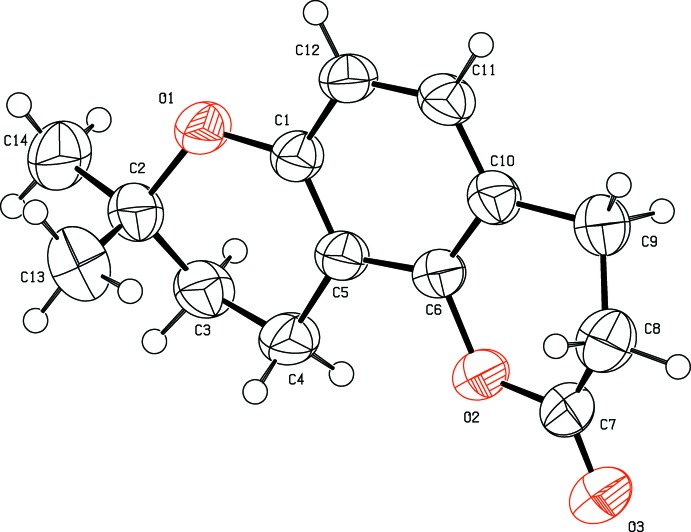
The mol­ecular structure of title compound, showing the atomic labelling. with displacement ellipsoids drawn at the 50% probability level

In the title compound, the three different fused rings comprising the mol­ecule (Fig. 1 ▸), are the central benzene ring (C1/C5–C12), the outer pyrone ring (O2/C6–C7) and the di­hydro­pyrane ring (O1/C1–C2), with dimethyl substituents attached at C3. The mean planes of these rings (O1/C1–C2 and O2/C6–C7) are inclined to the benzene plane by 6.20 (7) and 10.02 (8)°, respectively. The angles between the mean plane of the benzene ring and the four planar atoms of each pyran ring (O1/C1–C2) and (O2/C6–C10) are 3.0 (1)° (r.m.s. of the fitted atoms = 0.0092 Å) and 2.6 (1)° (r.m.s. of the fitted atoms = 0.0046 Å), respectively. Both rings are in half-chair conformations and atoms C2, C3, C7 and C8 deviate by 0.282, 0.446, 0.241 and 0.687 Å, respectively, from the plane through the other four essentially planar atoms of the respective pyran rings. These distortions of the di­hydro­pyran rings are probably the result of the ring flexibility and the presence of the methyl substituents. Experimental results from the title compound reveal that the photo-biological activity of the parent compound seselin has been diminished due to the formation of distorted half-chair conformations of the pyran rings on hydrogenation. The C6—C5—C1—O1 and C11—C10—C6—O2 torsion angles are almost the same *viz.* 178.44 (12) and 178.73 (14)°, respectively, indicating that these rings are coplanar. The destruction of photo-biological activity and change of conformation of the pyran rings of the title mol­ecule is considered to be due to the loss of the double bonds in seselin.

## Supra­molecular features   

**Figure 2:**
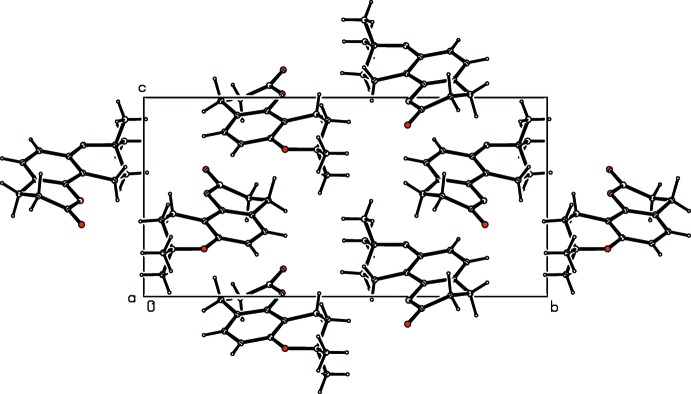
A view of the crystal packing in the unit cell of the title compound.

In the crystal, no formal hydrogen bonds are present but the mol­ecules exhibit very weak inter­molecular C—H⋯O inter­actions; none of these, however, can be considered as hydrogen bonds. Examples are: aromatic C8—H⋯O2^i^ (ring) [3.221 (2) Å] and methyl­ene C9—H⋯O3^i^ (carbon­yl) [3.412 (2) Å] inter­actions [symmetry code: (i) *x*, −*y* + 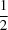, *z* − 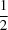], together with aromatic C12—H⋯O3^ii^ (ring) [3.598 (3) Å] and methyl­ene C8—H⋯O3^ii^ (carbon­yl) [3.593 (3) Å] inter­actions [symmetry code: (ii) *x* + 1, −*y* + 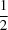, *z* − 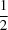], giving ‘ribbons’ extending along *a* through very weak head-to-tail 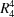
(8) ring motifs (Figs. 2 ▸ and 3 ▸). No π–π ring associations are present [minimum ring centroid separation = 4.654 (1) Å].

## Synthesis and crystallization   

The title compound was isolated as a colourless solid substance from the methanol extract of *T. stictocarpum* (in the local dialect, it is known as Aajmoda) by means of column chromatography over SiO_2_ gel by gradient elution with a binary mixed solvent system of hexane and ethyl acetate. It was purified by reverse phase high-pressure liquid chroma­tography (RP–HPLC) followed by crystallization to yield a colourless product. This compound was subjected to hydrogenation using Pd/C in a protic solvent (MeOH) at room temperature with continuous mechanical stirring overnight. The reaction product was worked up by the usual method to yield a crude product, which was was purified by column chromatography over SiO_2_ gel with gradient solvent elution to yield the pure title compound. Suitable crystals for X-ray diffraction analysis were obtained after recrystallization (×3) from ethyl acetate:hexane (1:4), by slow evaporation at room temperature. ^1^H NMR data (CDCl_3_, 200 MHz):δ_H_ 7.25 (*d*, 1H, *J* = 8.6 Hz, H-12), 6.68 (*d*, 1H, *J* = 8.6 Hz H-11), 2.40 (*t*, 1H, *J* = 6.6 Hz, H-4), 2.35 (*t*, 1H, *J* = 6.4 Hz, H-9), 2.26 (*t*, 2H, *J* = 6.4 Hz, H-8), 1.56 (*t*, 2H, *J* = 6.6 Hz, H-3), 1.50 (*s*, 3H, CH_3_, H-13), 1.54 (*s*, 3H, CH_3_, H-14).

## Database survey   

A search of the Cambridge Structural Database (CSD, Version 5.38, update November, 2016; Groom *et al.*, 2016 ▸) gave more than thirty five hits for both linear and angular pyran­ocoumarin (psoralene class) structures. They include four reports, CSD refcodes AMYROL [Kato, 1970 ▸: seselin (Amyrolin)]; AMYROL01 [Bauri *et al.*, 2006 ▸; seselin (redetermination)]; FUGVOS {Thailambal & Pattabhi, 1987 ▸: 2,3-dihy­droxy-9-hy­droxy-2(1-hy­droxy-1-methyl­eth­yl)-7*H*-furo[3,2-*g*]-[1]-benzo­pyran-7-one; bromo­hydroxy-seselin (Bauri *et al.*, 2017*a* ▸); di­bromo­mometh­oxy-seselin (DMS) (Bauri *et al.*, 2017*b* ▸)}, and a number of structures with various substituents at C3 and C4, many of which are natural products.

## Refinement   

Crystal data, data collection and structure refinement details are summarized in Table 1 ▸. All H atoms were located in difference-Fourier maps and the positional coordinates of all except the methyl H atoms were allowed to refine, with *U*_iso_(H) = 1.2*U*_eq_(C). Those on methyl groups were allowed to ride with C—H = 0.96 Å and with *U*_iso_(H) = 1.2*U*_eq_(C).

## Supplementary Material

Crystal structure: contains datablock(s) I. DOI: 10.1107/S205698901700932X/zs2379sup1.cif

Structure factors: contains datablock(s) I. DOI: 10.1107/S205698901700932X/zs2379Isup2.hkl

Supporting information file. DOI: 10.1107/S205698901700932X/zs2379Isup3.cml

CCDC reference: 1557474

Additional supporting information:  crystallographic information; 3D view; checkCIF report

## Figures and Tables

**Figure 3:**
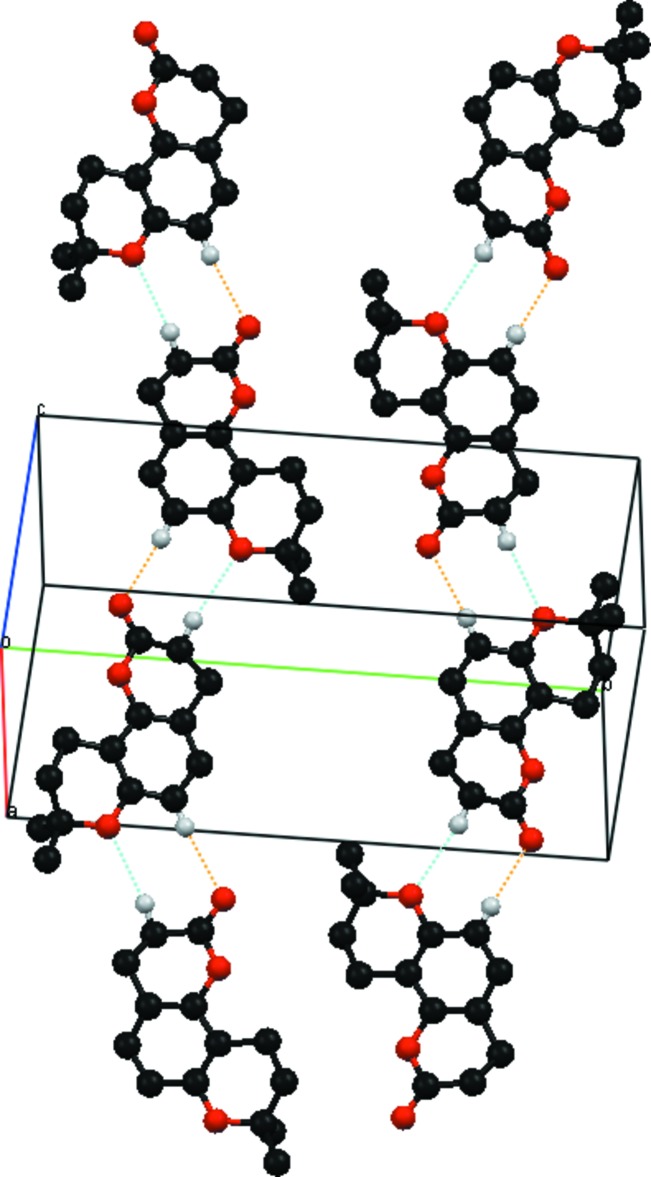
Part of the crystal structure, with weak C—H⋯O inter­actions shown as dashed lines. The most significant C—H⋯O_ring_ and *C*—H⋯O_carbon­yl_ inter­actions are shown as blue and orange dashed lines, respectively. Other H atoms have been omitted.

**Table 1:** Experimental details

Crystal data	
Chemical formula	C_14_H_16_O_3_
*M*_r_	232.27
Crystal system, space group	Monoclinic, *P*2_1_/*c*
Temperature (K)	299
*a*, *b*, *c* (Å)	7.282 (1), 18.445 (3), 9.144 (2)
β (°)	96.11 (3)
*V* (Å^3^)	1221.2 (4)
*Z*	4
Radiation type	Cu *K*α
μ (mm^−1^)	0.71
Crystal size (mm)	0.50 × 0.50 × 0.40

Data collection	
Diffractometer	Enraf–Nonius CAD-4
Absorption correction	ψ scan (North *et al.*, 1968 ▸)
*T*_min_, *T*_max_	0.717, 0.763
No. of measured, independent and observed [*I* > 2σ(*I*)] reflections	4924, 2187, 1954
*R*_int_	0.096
(sin θ/λ)_max_ (Å^−1^)	0.598

Refinement	
*R*[*F* ^2^ > 2σ(*F* ^2^)], *wR*(*F* ^2^), *S*	0.058, 0.151, 1.06
No. of reflections	2187
No. of parameters	187
H-atom treatment	H atoms treated by a mixture of independent and constrained refinement
Δρ_max_, Δρ_min_ (e Å^−3^)	0.32, −0.21

Computer programs: *CAD-4-PC* (Enraf–Nonius, 1996 ▸), *REDU4* (Stoe & Cie, 1987 ▸), *SHELXS97* and *SHELXL97* (Sheldrick, 2008 ▸) and *PLATON* (Spek, 2009 ▸).

## supplementary crystallographic information

### Crystal data

**Table:**

C_14_H_16_O_3_	*F*(000) = 496
*M**_r_* = 232.27	*D*_x_ = 1.263 Mg m^−^^3^
Monoclinic, *P*2_1_/*c*	Cu *K*α radiation, λ = 1.54180 Å
Hall symbol: -P 2ybc	Cell parameters from 25 reflections
*a* = 7.282 (1) Å	θ = 6.1–22.1°
*b* = 18.445 (3) Å	µ = 0.71 mm^−^^1^
*c* = 9.144 (2) Å	*T* = 299 K
β = 96.11 (3)°	Prism, colourless
*V* = 1221.2 (4) Å^3^	0.50 × 0.50 × 0.40 mm
*Z* = 4	

### Data collection

**Table:**

Enraf–Nonius CAD-4 diffractometer	1954 reflections with *I* > 2σ(*I*)
Radiation source: fine-focus sealed tube	*R*_int_ = 0.096
Graphite monochromator	θ_max_ = 67.1°, θ_min_ = 4.8°
ω/2θ scans	*h* = −8→8
Absorption correction: ψ scan (North *et al.*, 1968)	*k* = −22→0
*T*_min_ = 0.717, *T*_max_ = 0.763	*l* = −10→10
4924 measured reflections	3 standard reflections every 120 min
2187 independent reflections	intensity decay: 1.0%

### Refinement

**Table:**

Refinement on *F*^2^	Secondary atom site location: difference Fourier map
Least-squares matrix: full	Hydrogen site location: inferred from neighbouring sites
*R*[*F*^2^ > 2σ(*F*^2^)] = 0.058	H atoms treated by a mixture of independent and constrained refinement
*wR*(*F*^2^) = 0.151	*w* = 1/[σ^2^(*F*_o_^2^) + (0.0834*P*)^2^ + 0.1422*P*] where *P* = (*F*_o_^2^ + 2*F*_c_^2^)/3
*S* = 1.06	(Δ/σ)_max_ = 0.017
2187 reflections	Δρ_max_ = 0.32 e Å^−^^3^
187 parameters	Δρ_min_ = −0.21 e Å^−^^3^
0 restraints	Extinction correction: SHELXL97 (Sheldrick, 2008), Fc^*^=kFc[1+0.001xFc^2^λ^3^/sin(2θ)]^-1/4^
Primary atom site location: structure-invariant direct methods	Extinction coefficient: 0.089 (5)

### Special details

**Table:**

Geometry. All esds (except the esd in the dihedral angle between two l.s. planes) are estimated using the full covariance matrix. The cell esds are taken into account individually in the estimation of esds in distances, angles and torsion angles; correlations between esds in cell parameters are only used when they are defined by crystal symmetry. An approximate (isotropic) treatment of cell esds is used for estimating esds involving l.s. planes.
Refinement. Refinement of F^2^ against ALL reflections. The weighted R-factor wR and goodness of fit S are based on F^2^, conventional R-factors R are based on F, with F set to zero for negative F^2^. The threshold expression of F^2^ &gt; 2sigma(F^2^) is used only for calculating R-factors(gt) etc. and is not relevant to the choice of reflections for refinement. R-factors based on F^2^ are statistically about twice as large as those based on F, and R- factors based on ALL data will be even larger.

### Fractional atomic coordinates and isotropic or equivalent isotropic displacement parameters (Å^2^)

**Table:**

	*x*	*y*	*z*	*U*_iso_*/*U*_eq_	
C1	0.20282 (18)	0.18547 (7)	0.29558 (16)	0.0471 (4)	
C2	0.3391 (2)	0.07055 (8)	0.2387 (2)	0.0587 (5)	
C3	0.2859 (2)	0.04422 (8)	0.3851 (2)	0.0605 (5)	
H3A	0.288 (2)	−0.0078 (11)	0.378 (2)	0.073*	
H3B	0.382 (3)	0.0604 (10)	0.461 (2)	0.073*	
C4	0.0980 (2)	0.07214 (8)	0.4157 (2)	0.0616 (5)	
H4A	0.077 (3)	0.0653 (10)	0.515 (3)	0.074*	
H4B	−0.002 (3)	0.0455 (10)	0.349 (2)	0.074*	
C5	0.08030 (19)	0.15144 (7)	0.37942 (16)	0.0475 (4)	
C6	−0.05415 (18)	0.19413 (8)	0.43281 (16)	0.0477 (4)	
C7	−0.3262 (2)	0.18844 (9)	0.56020 (19)	0.0594 (5)	
C8	−0.3766 (2)	0.26087 (10)	0.4945 (3)	0.0713 (5)	
H8A	−0.433 (3)	0.2557 (11)	0.384 (3)	0.086*	
H8B	−0.459 (3)	0.2797 (12)	0.563 (3)	0.086*	
C9	−0.2123 (2)	0.30853 (9)	0.4840 (2)	0.0641 (5)	
H9A	−0.162 (3)	0.3240 (11)	0.594 (2)	0.077*	
H9B	−0.251 (3)	0.3509 (12)	0.429 (2)	0.077*	
C10	−0.0687 (2)	0.26811 (7)	0.41073 (18)	0.0515 (4)	
C11	0.0569 (2)	0.29965 (7)	0.32680 (18)	0.0534 (4)	
H11	0.050 (3)	0.3510 (10)	0.306 (2)	0.064*	
C12	0.1899 (2)	0.25945 (8)	0.26732 (18)	0.0522 (4)	
H12	0.277 (2)	0.2804 (10)	0.212 (2)	0.063*	
C13	0.2047 (3)	0.04668 (11)	0.1106 (2)	0.0818 (6)	
H13A	0.2463	0.0636	0.0205	0.098*	
H13B	0.0849	0.0665	0.1210	0.098*	
H13C	0.1975	−0.0053	0.1090	0.098*	
C14	0.5356 (3)	0.04849 (10)	0.2193 (3)	0.0811 (6)	
H14A	0.5689	0.0674	0.1280	0.097*	
H14B	0.5444	−0.0034	0.2187	0.097*	
H14C	0.6178	0.0676	0.2991	0.097*	
O1	0.34407 (14)	0.14972 (5)	0.23956 (13)	0.0580 (4)	
O2	−0.17164 (15)	0.15694 (6)	0.51821 (13)	0.0586 (4)	
O3	−0.41482 (18)	0.15412 (7)	0.63843 (17)	0.0787 (5)	

### Atomic displacement parameters (Å^2^)

**Table:**

	*U*^11^	*U*^22^	*U*^33^	*U*^12^	*U*^13^	*U*^23^
C1	0.0422 (7)	0.0445 (7)	0.0554 (8)	0.0013 (5)	0.0087 (6)	0.0009 (5)
C2	0.0557 (9)	0.0461 (8)	0.0758 (11)	0.0064 (6)	0.0141 (7)	−0.0035 (6)
C3	0.0594 (9)	0.0438 (8)	0.0792 (11)	0.0069 (6)	0.0112 (8)	0.0050 (7)
C4	0.0626 (9)	0.0441 (8)	0.0818 (11)	0.0018 (7)	0.0247 (8)	0.0087 (7)
C5	0.0455 (8)	0.0411 (7)	0.0567 (8)	−0.0012 (5)	0.0088 (6)	0.0012 (5)
C6	0.0449 (7)	0.0455 (8)	0.0538 (8)	−0.0038 (5)	0.0108 (6)	0.0005 (5)
C7	0.0495 (8)	0.0620 (9)	0.0695 (10)	−0.0067 (7)	0.0189 (7)	−0.0109 (7)
C8	0.0554 (9)	0.0700 (11)	0.0916 (14)	0.0063 (8)	0.0220 (9)	−0.0059 (9)
C9	0.0628 (10)	0.0520 (9)	0.0798 (12)	0.0073 (7)	0.0185 (8)	−0.0052 (8)
C10	0.0498 (8)	0.0440 (7)	0.0613 (8)	0.0017 (6)	0.0086 (7)	−0.0032 (6)
C11	0.0547 (8)	0.0388 (7)	0.0675 (9)	0.0004 (6)	0.0093 (7)	0.0035 (6)
C12	0.0496 (8)	0.0457 (8)	0.0627 (9)	−0.0036 (6)	0.0130 (7)	0.0063 (6)
C13	0.0920 (14)	0.0689 (11)	0.0833 (13)	0.0066 (9)	0.0041 (11)	−0.0151 (9)
C14	0.0697 (11)	0.0674 (11)	0.1112 (16)	0.0190 (8)	0.0325 (11)	0.0012 (10)
O1	0.0528 (6)	0.0465 (6)	0.0784 (8)	0.0039 (4)	0.0251 (5)	0.0034 (4)
O2	0.0566 (7)	0.0509 (6)	0.0723 (7)	−0.0033 (4)	0.0259 (6)	0.0017 (5)
O3	0.0724 (8)	0.0745 (8)	0.0967 (10)	−0.0108 (6)	0.0430 (7)	−0.0049 (6)

### Geometric parameters (Å, º)

**Table:**

C1—O1	1.3665 (17)	C7—C8	1.495 (3)
C1—C5	1.3869 (19)	C8—C9	1.496 (3)
C1—C12	1.390 (2)	C8—H8A	1.05 (2)
C2—O1	1.4608 (17)	C8—H8B	0.98 (2)
C2—C13	1.510 (3)	C9—C10	1.499 (2)
C2—C3	1.513 (3)	C9—H9A	1.08 (2)
C2—C14	1.516 (2)	C9—H9B	0.95 (2)
C3—C4	1.516 (2)	C10—C11	1.384 (2)
C3—H3A	0.96 (2)	C11—C12	1.377 (2)
C3—H3B	0.98 (2)	C11—H11	0.967 (18)
C4—C5	1.5025 (19)	C12—H12	0.936 (19)
C4—H4A	0.95 (2)	C13—H13A	0.9600
C4—H4B	1.03 (2)	C13—H13B	0.9600
C5—C6	1.385 (2)	C13—H13C	0.9600
C6—C10	1.382 (2)	C14—H14A	0.9600
C6—O2	1.3980 (17)	C14—H14B	0.9600
C7—O3	1.195 (2)	C14—H14C	0.9600
C7—O2	1.3576 (19)		
			
O1—C1—C5	122.89 (13)	C7—C8—H8B	101.7 (13)
O1—C1—C12	116.28 (12)	C9—C8—H8B	112.6 (13)
C5—C1—C12	120.81 (13)	H8A—C8—H8B	116.1 (17)
O1—C2—C13	108.01 (14)	C8—C9—C10	109.71 (14)
O1—C2—C3	108.93 (13)	C8—C9—H9A	106.9 (11)
C13—C2—C3	112.76 (16)	C10—C9—H9A	111.5 (11)
O1—C2—C14	104.25 (13)	C8—C9—H9B	108.9 (12)
C13—C2—C14	111.91 (17)	C10—C9—H9B	110.6 (13)
C3—C2—C14	110.55 (16)	H9A—C9—H9B	109.1 (16)
C2—C3—C4	112.07 (14)	C6—C10—C11	116.82 (13)
C2—C3—H3A	104.6 (12)	C6—C10—C9	118.19 (14)
C4—C3—H3A	111.9 (11)	C11—C10—C9	124.93 (13)
C2—C3—H3B	107.2 (12)	C12—C11—C10	121.81 (13)
C4—C3—H3B	111.0 (12)	C12—C11—H11	118.3 (12)
H3A—C3—H3B	109.7 (15)	C10—C11—H11	119.9 (12)
C5—C4—C3	110.34 (13)	C11—C12—C1	119.46 (13)
C5—C4—H4A	108.8 (11)	C11—C12—H12	122.5 (11)
C3—C4—H4A	111.9 (12)	C1—C12—H12	118.0 (11)
C5—C4—H4B	107.2 (11)	C2—C13—H13A	109.5
C3—C4—H4B	108.8 (11)	C2—C13—H13B	109.5
H4A—C4—H4B	109.6 (16)	H13A—C13—H13B	109.5
C6—C5—C1	117.26 (13)	C2—C13—H13C	109.5
C6—C5—C4	121.51 (13)	H13A—C13—H13C	109.5
C1—C5—C4	121.18 (13)	H13B—C13—H13C	109.5
C10—C6—C5	123.78 (13)	C2—C14—H14A	109.5
C10—C6—O2	121.60 (13)	C2—C14—H14B	109.5
C5—C6—O2	114.55 (12)	H14A—C14—H14B	109.5
O3—C7—O2	117.36 (16)	C2—C14—H14C	109.5
O3—C7—C8	126.07 (15)	H14A—C14—H14C	109.5
O2—C7—C8	116.39 (14)	H14B—C14—H14C	109.5
C7—C8—C9	112.78 (15)	C1—O1—C2	117.74 (11)
C7—C8—H8A	111.0 (11)	C7—O2—C6	121.53 (13)
C9—C8—H8A	103.2 (12)		
			
O1—C2—C3—C4	59.85 (19)	C5—C6—C10—C9	−175.60 (15)
C13—C2—C3—C4	−60.04 (19)	O2—C6—C10—C9	1.4 (2)
C14—C2—C3—C4	173.83 (14)	C8—C9—C10—C6	−32.6 (2)
C2—C3—C4—C5	−45.0 (2)	C8—C9—C10—C11	150.31 (17)
O1—C1—C5—C6	178.44 (12)	C6—C10—C11—C12	0.3 (2)
C12—C1—C5—C6	0.1 (2)	C9—C10—C11—C12	177.46 (14)
O1—C1—C5—C4	0.9 (2)	C10—C11—C12—C1	−2.0 (2)
C12—C1—C5—C4	−177.47 (15)	O1—C1—C12—C11	−176.63 (14)
C3—C4—C5—C6	−162.49 (15)	C5—C1—C12—C11	1.8 (2)
C3—C4—C5—C1	15.0 (2)	C5—C1—O1—C2	14.7 (2)
C1—C5—C6—C10	−2.0 (2)	C12—C1—O1—C2	−166.94 (14)
C4—C5—C6—C10	175.61 (15)	C13—C2—O1—C1	78.75 (18)
C1—C5—C6—O2	−179.11 (12)	C3—C2—O1—C1	−44.04 (18)
C4—C5—C6—O2	−1.5 (2)	C14—C2—O1—C1	−162.07 (15)
O3—C7—C8—C9	144.78 (19)	O3—C7—O2—C6	−176.80 (14)
O2—C7—C8—C9	−40.1 (2)	C8—C7—O2—C6	7.7 (2)
C7—C8—C9—C10	50.8 (2)	C10—C6—O2—C7	12.6 (2)
C5—C6—C10—C11	1.8 (2)	C5—C6—O2—C7	−170.16 (12)
O2—C6—C10—C11	178.73 (14)		
